# Bio-psycho-social factors’ associations with brain age: a large-scale UK Biobank diffusion study of 35,749 participants

**DOI:** 10.3389/fpsyg.2023.1117732

**Published:** 2023-06-09

**Authors:** Max Korbmacher, Tiril P. Gurholt, Ann-Marie G. de Lange, Dennis van der Meer, Dani Beck, Eli Eikefjord, Arvid Lundervold, Ole A. Andreassen, Lars T. Westlye, Ivan I. Maximov

**Affiliations:** ^1^Department of Health and Functioning, Western Norway University of Applied Sciences, Bergen, Norway; ^2^Norwegian Centre for Mental Disorder Research (NORMENT), Division of Mental Health and Addiction, Oslo University Hospital, University of Oslo, Oslo, Norway; ^3^Mohn Medical Imaging and Visualization Center (MMIV), Bergen, Norway; ^4^Department of Psychiatry, University of Oxford, Oxford, United Kingdom; ^5^LREN, Centre for Research in Neurosciences, Department of Clinical Neurosciences, Lausanne University Hospital, University of Lausanne, Lausanne, Switzerland; ^6^Faculty of Health, Medicine and Life Sciences, Maastricht University, Maastricht, Netherlands; ^7^Department of Psychiatric Research, Diakonhjemmet Hospital, Oslo, Norway; ^8^Department of Psychology, University of Oslo, Oslo, Norway; ^9^Department of Radiology, Haukeland University Hospital, Bergen, Norway; ^10^Department of Biomedicine, University of Bergen, Bergen, Norway; ^11^KG Jebsen Centre for Neurodevelopmental Disorders, University of Oslo, Oslo, Norway

**Keywords:** brain age, age prediction, magnetic resonance imaging, diffusion MRI, health, cognition, brain variability

## Abstract

Brain age refers to age predicted by brain features. Brain age has previously been associated with various health and disease outcomes and suggested as a potential biomarker of general health. Few previous studies have systematically assessed brain age variability derived from single and multi-shell diffusion magnetic resonance imaging data. Here, we present multivariate models of brain age derived from various diffusion approaches and how they relate to bio-psycho-social variables within the domains of sociodemographic, cognitive, life-satisfaction, as well as health and lifestyle factors in midlife to old age (*N* = 35,749, 44.6–82.8 years of age). Bio-psycho-social factors could uniquely explain a small proportion of the brain age variance, in a similar pattern across diffusion approaches: cognitive scores, life satisfaction, health and lifestyle factors adding to the variance explained, but not socio-demographics. Consistent brain age associations across models were found for waist-to-hip ratio, diabetes, hypertension, smoking, matrix puzzles solving, and job and health satisfaction and perception. Furthermore, we found large variability in sex and ethnicity group differences in brain age. Our results show that brain age cannot be sufficiently explained by bio-psycho-social variables alone. However, the observed associations suggest to adjust for sex, ethnicity, cognitive factors, as well as health and lifestyle factors, and to observe bio-psycho-social factor interactions’ influence on brain age in future studies.

## 1. Introduction

Developmental trajectories of brain morphology are informative signaling markers of health. For example, significant deviations from normative morphology values can signify the presence or development of disease (Marquand et al., 2019; Remiszewski et al., 2022). Based on the idea that a general normative pattern could describe brain trajectories, the concept of brain age has been introduced. Here, different brain features are used to predict individuals’ age. The difference between such predicted age and chronological age, the brain age gap (BAG), has the potential as a general health biomarker, sensitive to various neurological, neuropsychiatric, and neurodegenerative disorders (Kaufmann et al., 2019; Cole, 2020; Rokicki et al., 2021). Brain age can be derived using different imaging modalities. Structural and diffusion MRI (dMRI) have shown high prediction accuracy (e.g., Cole, 2020; Beck et al., 2022b; Chen et al., 2022; Leonardsen et al., 2022; Sone et al., 2022). Different dMRI-derived parameters allow one to describe multiple changes in WM micro-structure using various diffusion-weighted approaches. Such dMRI measures provide invaluable information about WM architecture at the micrometer scale and can be associated with macroscopic outcomes. The most popular dMRI approach, diffusion tensor imaging (DTI), is often used to describe WM organization (Basser et al., 1994). However, methodological advances and newer diffusion approaches may provide more meaningful bio-physical information (Novikov et al., 2019), thereby increasing the power of cross-validation of findings and their comparability with other clinical markers (Billiet et al., 2015; Kamagata et al., 2020; Beck et al., 2021; Wood et al., 2022).

**Figure 1:**
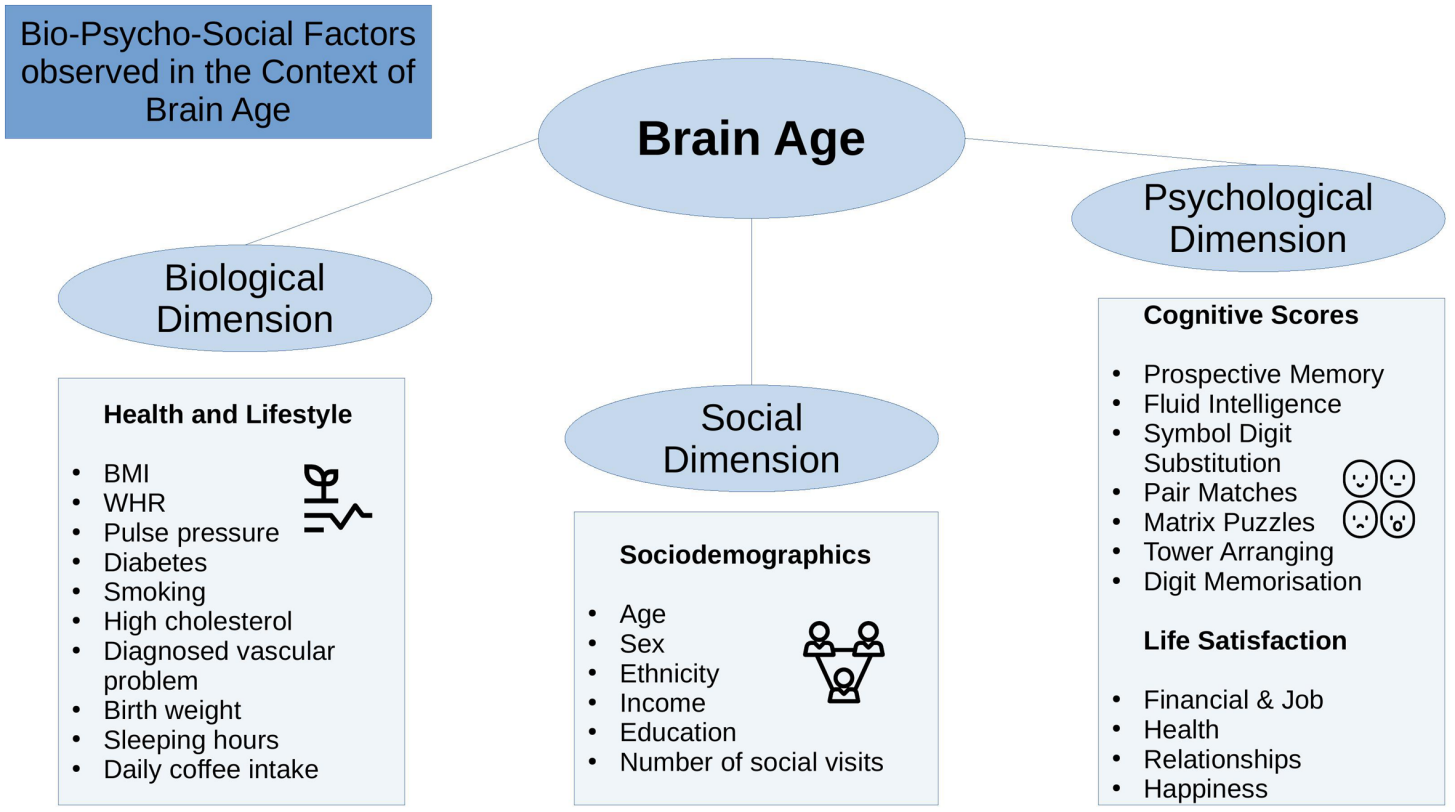
Overview of the variables used.

The bio-psycho-social model (Engel, 1977) strives for a holistic perspective on medical research to understand health and disease by integrating information on biological, psychological, and social factors (e.g., Ghaemi, 2009; Wade and Halligan, 2017). Brain age can be utilized in this context as an indicator of general health (Kaufmann et al., 2019), using the different levels of observation (bio-psycho-social) to describe brain age relationships with different phenotypes. While there are some studies providing evidence for brain age associations with bio-psycho-social factors, including demographic, biomedical, lifestyle, cognitive, and behavioral factors (Cole, 2020; de Lange et al., 2021; Beck et al., 2022b; Leonardsen et al., 2022; Sone et al., 2022), it remains unclear whether brain age derived from different diffusion approaches relates differentially to sociodemographic, health, life-satisfaction, and cognitive factors (Figure 1), and what the qualities of such relationships are.

While brain age is a proxy for different health-related processes, similar to various bio-psycho-social factors, it remains largely unclear how brain age and bio-psycho-social factors associate. It is hence necessary to increase our understanding and the interpretability of brain age by observing the associations of common phenotypes with brain age. There are large differences in the usage of underlying data and machine learning approaches applied to the data for brain age predictions (Franke and Gaser, 2019). Practical effects of such differences, for example, on phenotype associations, have yet to be systematized to better interpret brain age and BAG. The bio-psycho-social approach (Engel, 1977) lends itself to categorizing phenotypes into concrete groups. Within the groups, phenotype associations with brain age in general can be considered in addition to differences in underlying data used to calculate brain age. Here, we limit our investigations to dMRI-derived brain age to examine brain age relationships with bio-psycho-social factors specific to WM. WM has repeatedly been shown to change throughout ageing and to relate to different bio-psycho-social variables (Le Bihan and Iima 2015; Beck et al., 2021, 2022a,b). Although comparisons of single MRI modality predictions from either T1-weighted or dMRI depend on the model selection and observed parameter choice (Niu et al., 2019; Rokicki et al., 2021), models using T1-weighted and dMRI features show comparable age prediction performance (Cole, 2020; de Lange et al., 2021). However, phenotype-WM-brain-age relationships require still further examination. Using different diffusion approaches in this context will not only help extend commonly used diffusion tensor imaging by giving reference values to other brain age derived from other WM metrics but also provide a clearer understanding of WM-phenotype associations.

Diffusion MRI can describe various biological processes by providing markers of brain tissue changes across the lifespan (Beck et al., 2021). These markers are not only heritable (Elliott et al., 2018) but also indicative of health, for example, by being associated with psychiatric and neurological disorders, addiction, stroke (Le Bihan and Iima 2015), or cardiovascular health (Beck et al., 2022b). Various diffusion metrics that have previously been related to cognitive and mental health traits have also shared genetic underpinnings with cognitive and mental health phenotypes (Zhao et al., 2021). The biological underpinnings of dMRI markers become particularly apparent in WM abnormalities observed in severe mental disorders, including schizophrenia (Cetin-Karayumak et al., 2020) or bipolar disorder (Houenou et al., 2007). Furthermore, dMRI-derived brain age is higher in people showing accumulations of cardiometabolic risk and markers of adipose tissue distribution (Beck et al., 2022a,b). Such associations between phenotypes and brain age can also be observed when comparing high to low socioeconomic status (SES) groups, where low SES individuals have lower WM integrity (Pavlakis et al., 2015; Shaked et al., 2019).

Furthermore, dMRI offers both single and multi-shell approaches and various meaningful metrics describing white matter microstructure (Jensen et al., 2005; Fieremans et al., 2011; Kaden et al., 2016a,b; Reisert et al., 2017), which serve as a good basis for brain age estimations (Beck et al., 2021; Korbmacher et al., 2022) exploiting biophysically meaningful parameters of brain tissue in contrast to general measures such as grey/white volume or thickness.

While there are various diffusion models offering a plethora of metrics, most efforts have focussed on DTI which provides fractional anisotropy, which decreases, and radial, axial, and mean diffusivity, which increase over the lifetime, respectively, indicating a loss of structural integrity (Westlye et al., 2010; Behler et al., 2021). Advanced diffusion approaches also examine structural integrity, but adding further detail such as the differentiation between intra-and extra-axonal space, parametrization of extra-axonal diffusivity, and axonal bundle distribution (Jensen et al., 2005; Fieremans et al., 2011; Kaden et al., 2016a,b; Reisert et al., 2017).

Differences in brain age-phenotype relationships can be expected when varying dMRI approaches, as varying underlying dMRI approaches will also produce variability in brain age predictions (see Beck et al., 2021; Korbmacher et al., 2022), potentially due to measuring different bio-physical processes (Jensen et al., 2005; Fieremans et al., 2011; Kaden et al., 2016a,b; Reisert et al., 2017). These potential differences become important when attempting to generalize findings on brain age across the literature and setting standards for brain age predictions. However, to what extent age predictions based on single and multi-shell dMRI approaches relate differentially to phenotypes requires further investigation. Hence, comparing dMRI-based brain age predictions can be fruitful, not only when expanding current efforts of examining brain age associations with phenotypes but also by investigating whether differences in the underlying data can influence relationships of brain age with bio-psycho-social variables.

State-of-the-art conceptualizations of health, such as the bio-psycho-social model (Engel, 1977), recommend considering various domains or levels of explanation when assessing health outcomes, such as brain age. In that sense, brain age can related to different biological, psychological and social factors. The extent of the relationships are important as they can inform on which bio-psycho-social factors lead to better compared to worse brain or general health. Beyond validating brain age as a concept, this can directly improve our understanding of health. To date, brain age is usually calculated from a large range of MRI features. The resulting brain age estimate is then usually predicted from single variables of interest while controlling for sex and age (e.g., Cole, 2020; Leonardsen et al., 2022). However, cumulative and synergy effects can be expected to partly explain health, which has, for example, been shown for cardiometabolic risk factors explaining brain age (see Beck et al., 2022a,b). Hence, we group available phenotypes that have previously been found influential for health (Figure 1) into health and lifestyle factors, representing the biological dimension of the bio-psycho-social model, respectively (Erhardt, 2009; Ning et al., 2020; Gill et al., 2021; Vidal-Pineiro et al., 2021; Beck et al., 2022a,b; Pham et al., 2022). Life satisfaction factors and cognitive factors represent the psychological dimension, and sociodemographic factors the social dimension of the bio-psycho-social model, respectively.

Generally, explaining brain age variance is required to further our understanding of brain age and its multivariate relationship with different phenotypes influencing physiology directly or indirectly. We, therefore, extend previous work by explaining variance in brain age by combining sets of bio-psycho-social variables into domains of sociodemographic, health, life satisfaction, and cognitive factors (Figure 1) to assess their associations with brain age. In addition to exploring associations of bio-psycho-social variables with dMRI-based brain age, we differentiate between diffusion approaches used for brain age predictions and exame the consistency across diffusion approaches. Previous findings revealed weak associations of various phenotypes with brain age in the UK Biobank (e.g., Smith et al., 2019; Cole, 2020). Hence, we expect only small proportions of the variance in brain age to be predicted by bio-psycho-social variables. We also hypothesize that factors directly representing or impacting physiology are more predictive of brain age than those which impact physiology only indirectly. Thus, health factors are presumed to be more predictive of brain age than sociodemographic, cognitive, and life satisfaction factors. Finally, we expect some variability in these associations to be due to the underlying diffusion approach, as different WM properties are also expected to be differentially related to phenotypes. We may move brain age closer to the clinical utility by furthering our understanding of brain age.

## 2. Methods

### 2.1. Sample characteristics

The sample used has been described elsewhere (Korbmacher et al., 2022). In brief, the UK Biobank (UKB) (Sudlow et al., 2015) diffusion MRI data consisted of *N* = 42,208 participants. We excluded subjects who withdrew their informed consent (up to 22nd of February 2022) or with an ICD-10 diagnosis from categories F, G, I, or stroke from the general health assessment (Field 42,006; excluded: *N* = 3,521). We also excluded data that did not pass our quality control (*N* = 2,938) using the YTTRIUM method (Maximov et al., 2021). In brief, YTTRIUM converts diffusion scalar metric into 2D format using a structural similarity extension (Wang et al., 2004) of each scalar map to their mean image to create a 2D distribution of image and diffusion parameters. Quality check is based on 2 step clustering algorithm in order to identify subjects out of the main distribution. Our final sample consisted of 35,749 healthy adults. For an overview of demographics and the bio-psycho-social variables included in this study and their relationship with brain age see Table 1.

**Table 1:** Overview of the predictors used in the bio-psycho-social models.

Model	Predictors
Model 1. Baseline	Age Sex Scanner site*
Model 2. Socio-demographics	Age Sex Scanner site* Ethnicity Income Education Nb of social visits
Model 3. Cognitive Scores	Age Sex Scanner site* Prospective memory Fluid intelligence Symbol digit substitution Pair matches Matrix puzzles Tower arranging Digit memorization
Model 4. Life satisfaction	Age Sex Scanner site* Financial and job satisfaction Friend and family relation satisfaction Happiness
Model 5. Health and lifestyle	Age Sex Scanner site* BMI WHR Pulse pressure Diabetes Smoking High cholesterol Diagnosed vascular disorder Birth weight Sleeping hours Daily coffee intake Alcohol drinker

*Random effect.

### 2.2. MRI acquisition, diffusion post-processing, and TBSS analysis

UKB MRI data acquisition procedures are described elsewhere (Sudlow et al., 2015; Miller et al., 2016; Alfaro-Almagro et al., 2018). Briefly, single and multi-shell data were acquired at four different locations using identical scanners: 3 T Siemens Skyra, with a standard 32-channel head coil and key diffusion parameters being MB = 3, *R* = 1, TE/TR = 92/3600 ms, PF 6/8, fat sat, b = 0 s/mm^2^ (5x + 3× phase-encoding reversed), *b* = 1,000 s/mm^2^ (50×), b = 2,000 s/mm^2^ (50×) (Alfaro-Almagro et al., 2018).

We obtained access to the raw diffusion data and pre-processed the data using an optimized pipeline as described by Maximov et al. (2019). The pipeline includes corrections for noise (Veraart et al., 2016), Gibbs ringing (Kellner et al., 2016), susceptibility-induced and motion distortions, and eddy current artifacts (Andersson and Sotiropoulos, 2016). Isotropic 1 mm^3^ Gaussian smoothing was carried out using FSL’s (Smith et al., 2004; Jenkinson et al., 2012) *fslmaths*. Employing the multi-shell data, Diffusion Tensor Imaging (DTI), Diffusion Kurtosis Imaging (DKI) (Jensen et al., 2005) and White Matter Tract Integrity (WMTI) (Fieremans et al., 2011) metrics were estimated using Matlab 2017b code.^1^ Spherical mean technique SMT (Kaden et al., 2016b), and multi-compartment spherical mean technique (mcSMT) (Kaden et al., 2016a) metrics were estimated using original code^2^ (Kaden et al., 2016a,b). Estimates from the Bayesian Rotational Invariant Approach (BRIA) were evaluated by the original Matlab code^3^ (Reisert et al., 2017).

Previous advances observing age-dependent WM changes have largely focused on single-shell diffusion, such as DTI with DTI-derived metrics being fractional anisotropy (FA), and axial (AD), mean (MD), and radial (RD) diffusivity, all being highly sensitive to age (Westlye et al., 2010; Cox et al., 2016; Beck et al., 2021). More recently developed multi-shell diffusion approaches which extend the space of derivable diffusion metrics appear more sensitive to brain changes and sex differences (Lawrence et al., 2021), and at the same time less sensitive to motion artefacts than single-shell models (Pines et al., 2020). Newer approaches are (1) BRIA, as an alternative to not rely on fiber orientation but rotation invariant feature (Reisert et al., 2017), (2) DKI, a method tackling the problem of non-Gaussian diffusion (Jensen et al., 2005); (3) WMTI, which extends DKI by calculating inter and extra-axonal features (Fieremans et al., 2011); and (4) SMT (Kaden et al., 2016b) and (5) mcSMT, which factor out neurite orientation to give a better estimate of microscopic diffusion anisotropy (Kaden et al., 2016a). The selection of diffusion models was dictated by a few practical reasons. There are two conventional approaches (DTI and DKI) describing the general WM changes. As a result, these approaches are expected to be sensitive to a broad range of aging-related effects associated with WM maturation (Westlye et al., 2010; Yap et al., 2013). Advanced dMRI approaches enable more detailed quantification associated with age in a different manner (Cox et al., 2016; Beck et al., 2021). Diffusion modelling relies on biophysically motivated assumptions such as the axon bundle distribution (WMTI) or attempts to suppress such kind of parameters (SMT and SMT mc). Another modelling option are Bayesian rotation invariants (BRIA), providing multiple measures of WM but depending on efficacy of initial Bayesian simulations. All together, these approaches allow us to indirectly verify the stability and reliability of diffusion assumptions in brain-age prediction on their own and in comparison to each other, or to determine similarity among scalar metrics appearing in several diffusion approaches.

In total, we obtained 28 metrics (Supplementary Table S1) from six diffusion modeling approaches (DTI, DKI, WMTI, SMT, mcSMT, and BRIA). To normalize all metrics, we used tract-based spatial statistics (TBSS) (Smith et al., 2006) as part of FSL (Smith et al., 2004; Jenkinson et al., 2012). In brief, initially, all FSL BET-extracted (Smith, 2002) FA images were aligned to MNI space using non-linear transformation (FNIRT) (Jenkinson et al., 2012). Subsequently, we derived the mean FA image and the related mean FA skeleton. Each diffusion scalar map was projected onto the mean FA skeleton using the standard TBSS procedure. To provide a quantitative description of diffusion metrics we evaluated averaged values over the skeleton and two WM atlases, namely the Johns Hopkins University (JHU) atlas (Mori et al., 2005) and the JHU tractography atlas (Hua et al., 2008; see Supplementary Table S2 for an overview). Finally, we obtained 20 WM tracts and 48 regions of interest (ROIs) based on a probabilistic WM atlas (JHU) (Hua et al., 2008) for each of the 28 metrics, including the mean skeleton values. Altogether, we derived 1,932 features per individual [28 metrics * (48 ROIs +1 skeleton mean + 20 tracts)]; see Supplementary Table S1 for metrics and Supplementary Table S2 for regions and tracts.

### 2.3. Brain age predictions

We computed brain age predictions derived from 8 different models including the six diffusion approaches, their whole-brain average scores (mean multimodal), and a model combining the six diffusion approaches and their whole-Brian average scores (full multimodal). Each of the six diffusion approaches details WM features based on differing modelling assumptions and were assumed to provide unique brain age scores. Whole-brain average scores for each of the six diffusion approaches’ metrics were investigated on their own to further our understanding of spacial specificity. Finally, previous results (de Lange et al., 2020b; Beck et al., 2021, 2022b) provide clear evidence of strong age prediction performance when combining diffusion metrics. We hence included a model combining all diffusion approaches’ metrics and their whole-brain average scores to compare whether there are differences in multimodal to single diffusion approaches’ brain-age-phenotype associations.

Brain age was predicted using the XGBoost tree-boosting algorithm (gradient boosting tree) implemented in Python (v3.7.1), being a highly effective algorithm for tabular data (Chen and Guestrin, 2016). From the total included sample (*N* = 35,749), we used 10% (N = 3,575) for hyperparameter tuning on a data set containing data from all diffusion approaches (i.e., full multimodal data with 1,932 features/parameters) using 5-fold cross-validation (after estimating an optimal hyperparameter tuning set size; Korbmacher et al., 2022). The considered hyperparameters for the randomized grid search were (1) learning rate with a range of 0.01–0.3 and steps of 0.05, (2) maximum layers/depth with a range of 3–6 and steps of 1, and (3) number of trees with a range of 100–1,000 and steps of 50. The resulting hyperparameters (learning rate = 0.05, max layers/depth = 3, and the number of trees = 750) were then used in a 10-fold cross-validation applied to the test set (*N* = 32,174). Cross-validation was used to leverage the full sample size and to calculate the uncertainty around the estimates (for such see Korbmacher et al., 2022). The cross-validation procedure was executed using each of the six diffusion approaches’ metrics, whole-brain averaged metrics for all approaches (mean multimodal model), and finally a combination of all approaches and the whole-brain average scores (full multimodal model), resulting in eight brain age models (see Supplementary Table S1 for dMRI approach-specific metrics). Each of these brain ages were used in the analyses. See Supplementary Figure S1 for an overview of the brain age models and the following modelling of these predictions from the bio-psycho-social models.

### 2.4. Statistical analyses

All statistical analyses were carried out using Python, version 3.7.1 and R, version 4.2.0^4^ using test data set (*N* = 32,174). These analyses focused on the associations between brain age and (1) demographics, (2) social factors, (3) cognitive test scores, (4) life satisfaction, and (5) health and lifestyle factors (with weight on cardiometabolic factors). For detailed information on how variables were extracted and coded see Supplementary Table S3. First, we calculated the first principal component of bio-psycho-social factors’ by grouping numeric variables of each of the 5 domains (demographics, social factors, cognitive tests scores, life satisfaction, and health and lifestyle factors), using scaling and the number of allowed components equal to the number of variables included. We then examined the first components’ associations with brain age. Second, we examined to which extend multivariate models (as specified in 2.4.1) explain brain age from the factors of the five bio-psycho-social domains. Finally, we tested whether our findings would be influenced by analyzing data separately for males and females, and present bi-variate relationships between multimodal brain age and single bio-psycho-social variables.

For bi-variate relationships between bio-psycho-social factors and full multimodal brain age, we adjusted *p*-values for multiple comparison using Bonferroni correction, dividing the alpha-level by twenty-five (α/25), the number of bi-variate associations observed. For multivariate relationships we divided alpha by eight (α/8), the number of brain age models used. Furthermore, the coefficient of determination describing the proportion of variance explained (*R*^2^) will be presented as marginal *R*^2^, referring to variance explained by fixed effects, and conditional *R*^2^, referring to both fixed and random effects variance explained.

#### 2.4.1. Bio-psycho-social models explaining brain age

We used linear mixed effects models with the random intercepts at the level of scanner site to explain changes in brain age from socio-demographics, cognitive test scores, life satisfaction (self-assessment), and health and lifestyle factors. The presented models were used in two different ways: first, with the principal component of the model-specific bio-psycho-social factors replacing the respective bio-psycho-social factors, and second using all eight brain ages from the different diffusion approaches on with the models. For an overview of the predictors in the multivariate bio-psycho-social models used see Table 1.

We established the following models to compare:

(1) A baseline model capturing the relationship of age, sex, the age-sex interaction, and scanner site with brain age. This baseline model was selected as predicted age is expected to be largely reflected by chronological age. However, also sex (e.g., Rokicki et al., 2021), and scanner site (here, Bristol, Cheadle, Newcastle, Reading) and prediction bias (e.g., Jirsaraie et al., 2022) have been shown to be influential for brain age. Using a baseline model and additional models for comparison had the goal to estimate added variance explained by the bio-psycho-social models above and beyond the baseline mode (Bollen, 1989). Additionally, predictors within these bio-psycho-social models were observed individually (bivariate compared to multivariate relationships with brain age). Model comparison to a baseline model (instead of a null model) is important in this context as brain age is sensitive to age, sex and scanner site (de Lange and Cole, 2020; Rokicki et al., 2021; Jirsaraie et al., 2022). Hence, instead of using a null model which does not contain much information, we used the following model as a reference point for further model comparison:


brainage=sex+age+sex∗age+site

(2) A sociodemographic model additionally included ethnic ancestry (binary yes/no self-reported white European; for additional information sample groupings by ethnicity see Supplementary Table S4), average annual total household income before tax (coded as continuous variable 1–5, with low <£18,000 to high income >£100,000), and higher education (binary yes/no self-report of having obtained higher education) relative to the baseline model.


$$\begin{array}{c} brainage=sex+age+sex*age+ethnicity\\ +income+education+site \end{array}$$

(3) A cognitive model testing how non-verbal cognitive abilities add to the baseline model (overview: Fawns-Ritchie and Deary, 2020). We limited the selection of cognitive variables to non-verbal assessment measures to reduce the parameter space of cognitive variables and as non-verbal assessment scores have been found to associated with dMRI metrics throughout the lifespan (e.g., Sullivan and Pfefferbaum, 2006; Sasson et al., 2010; McPhee et al., 2019; Parikh et al., 2021). Namely, the number of matrix puzzles solved (matrixS) testing non-verbal reasoning using COGNITO Matrices, tower arranging correctly solved (towerS) testing executive function using the Delis-Kaplan Executive Function System Tower Test, prospective memory (memory) assessed with the Rivermead Behavioural Memory Test, fluid intelligence (intel) from the UKB own Fluid IQ test, digits remembered (digits) from the Symbol Digit Modalities Tests, and the mean number of incorrect pair matches (IPM) across trail A and B assessing visual declarative memory using the Wechsler Memory Scale IV Designs I and Designs II. Correlations were small to moderate (*r_max_* = 0.41) with the variance inflation factor (VIF) indicating low levels of multicollinearity (Supplementary Figure S2).


$$\begin{array}{c} brainage=sex+age+sex*age+matrixS+towerS\\ +memory+intel+digits+IPM+site \end{array}$$

(4) A life satisfaction model that additionally included job satisfaction (jobS), financial satisfaction (financeS), overall health rating (healthR), health satisfaction (healthS), family relation satisfaction (famS), happiness, friend relationship satisfaction (friendS) relative to the baseline model. Some of the model features were highly correlated (*r_max_* = 0.65), yet VIF values indicated low levels of multicollinearity (Supplementary Figure S3).


$$\begin{array}{c} brainage=sex+age+sex*age+jobS\\ +financeS+healthR+healthS\\ +famS+friendS+happiness+site \end{array}$$

(5) A health and lifestyle model testing how body mass index (BMI), pulse pressure (Ppressure: the difference between systolic and diastolic blood pressure), waist-to-hip-ratio (WHR), binary smoking status, binary diabetes diagnosis (both type I and II), binary high cholesterol (chol), binary diagnosed vascular problem (DVP), birth weight (Bweight), sleeping hours, and daily coffee intake (coffee) add to the baseline model, with only BMI and WHR showing a moderate correlation *r* = 0.42, but all other correlations being small *rs* < 0.16, with VIF values indicating only low levels of multicollinearity (Supplementary Figure S4).


$$\begin{array}{c} brainage=sex+age+sex*age+BMI+WHR+Ppressure\\ +diabetes+smoking+chol+DVP\\ +Bweight+coffee+site \end{array}$$

#### 2.4.2. Follow-up and quality control analyses and single bio-psycho-social factor associations with multimodal brain age

Previous research showed sex differences in brain age, suggesting sex separate analyses (Rokicki et al., 2021). Hence, we conducted the analyses described in 2.4.1 separately for males and females.


brainage=age+site+biopsychosocialfactors

To examine the contributions of single bio-psycho-social variables to explaining WM brain age, linear mixed models were used to observe bio-psycho-social variable associations with brain age when controlling for age and sex with scanner site as a random factor. In other words, different from 2.4.1, we applied one model per bio-psycho-social factor. For simplicity, this analysis step only considered the best brain age predictions from the multimodal model including the metrics of all diffusion approaches (Korbmacher et al., 2022).


$$\begin{array}{c} brainage=sex+age+sex*age+site\\ +singlebiopsychosocialfactor \end{array}$$

Each model was then compared to a model not including the respective bio psycho social variable:


brainage=sex+age+sex∗age+site

## 3. Results

### 3.1. Linear mixed effect models explaining brain age gap from bio-psycho-social factors

We ran the proposed five baseline and bio-psycho-social models with the first principal component (PC) of the numeric predictors from each of the models showing a small proportion of the variance in brain age uniquely explained by the principal components (*R*^2^ < 1%; Supplementary Table S5), with differences between these models and respective baseline models yet being highly significant (Supplementary Table S6).

**Figure 2:**
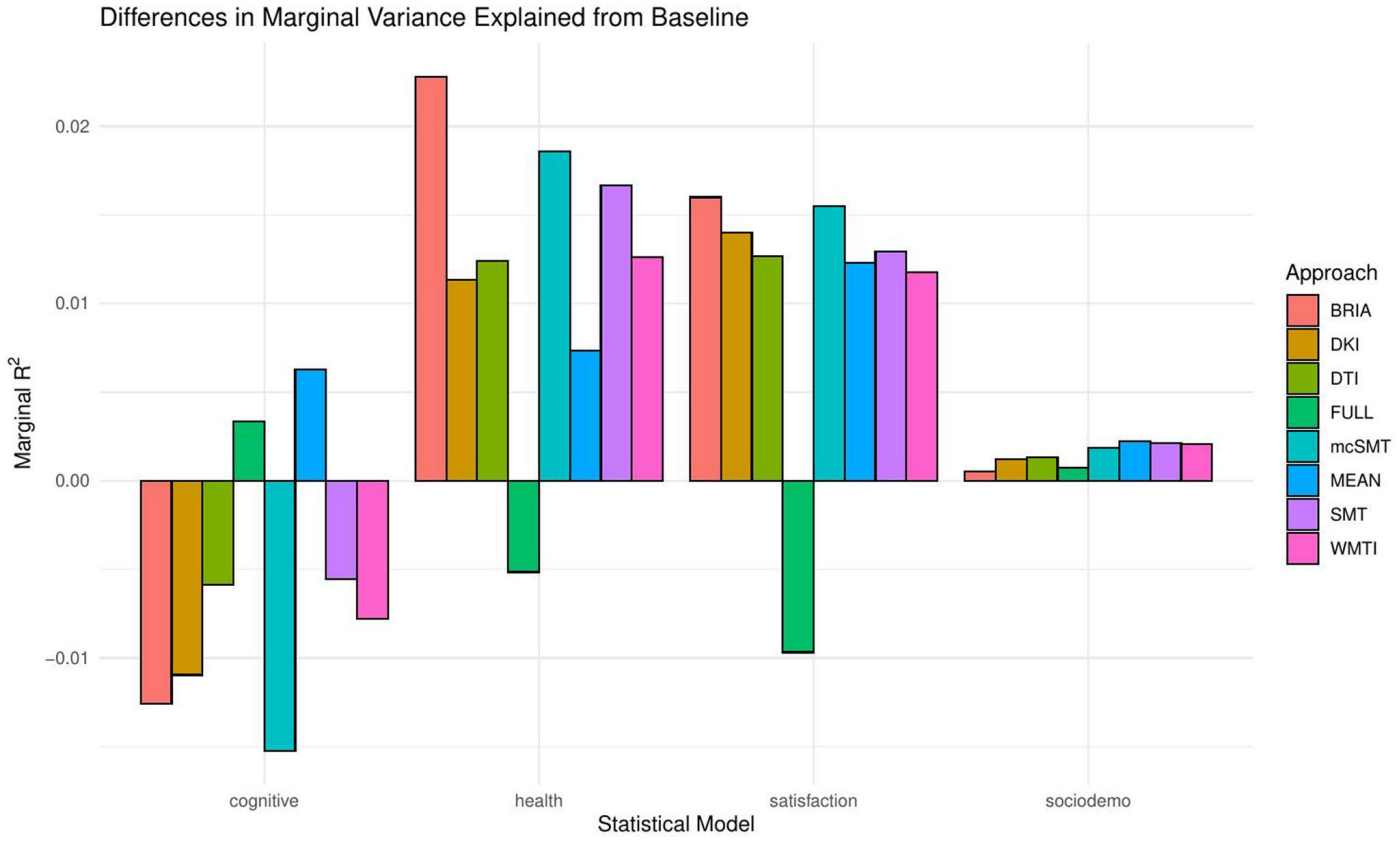
Marginal *R*^2^ values for statistical models across diffusion approaches.

**Figure 3:**
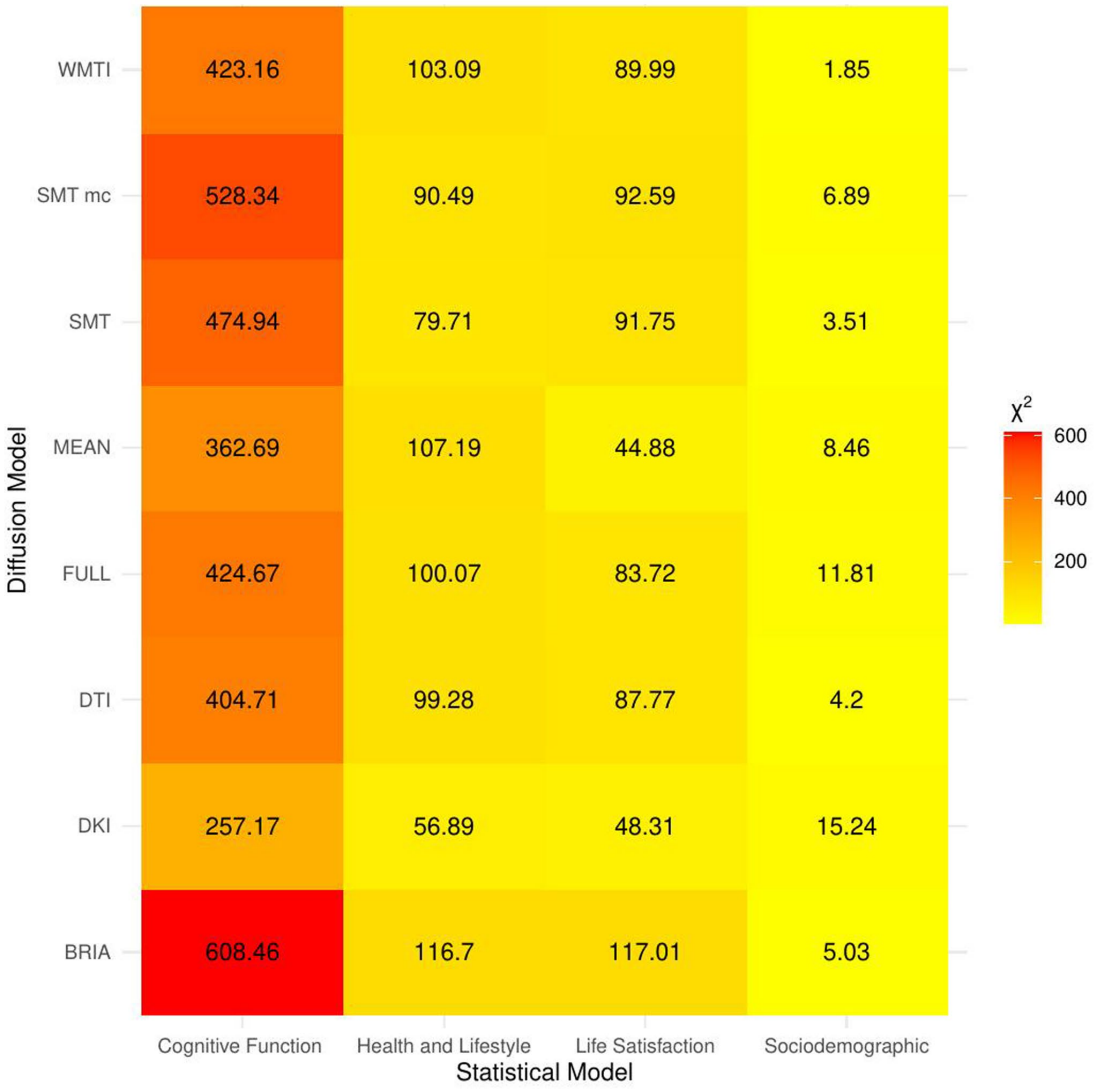
Overview of comparison of bio-psycho-social statistical models with baseline models. The figure presents *χ*^2^ values for each of the bio-psycho-social statistical models for each diffusion approach tested against the baseline model. Note that only values of χ^2^ > 11 were significant (*p* < 0.05).

When including bio-psycho-social factors instead of their PCs and comparing baseline to models 2–5, a larger proportion of both marginal or conditional variance in brain age could be uniquely explained by bio-psycho-social variables (marginal and conditional *R*^2^ < 0.03; Figure 2 and Supplementary Table S7). Model comparisons showed that, with the exception of socio-demographic factors, bio-psycho-social models explained significantly more variance in brain age than the baseline model (with age, sex, and age-by-sex interaction as fixed and scanner site as random effect), irrespective of the diffusion approach used to calculate brain age (*ps* < 0.01; Figure 3). Differences between this uniquely explained marginal variance were small across diffusion approaches (Figure 2).

**Figure 4:**
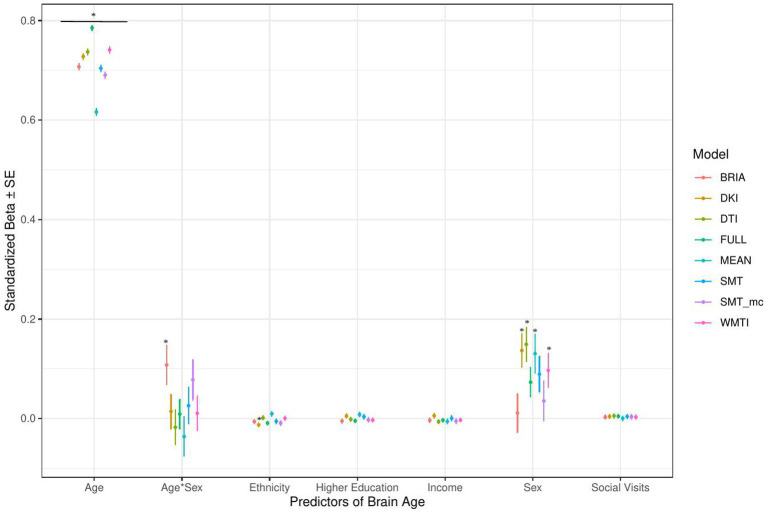
Sociodemographic model predictors’ standardized beta-values with standard error. *Indicates Bonferroni-corrected *p* < 0.05.

**Figure 5:**
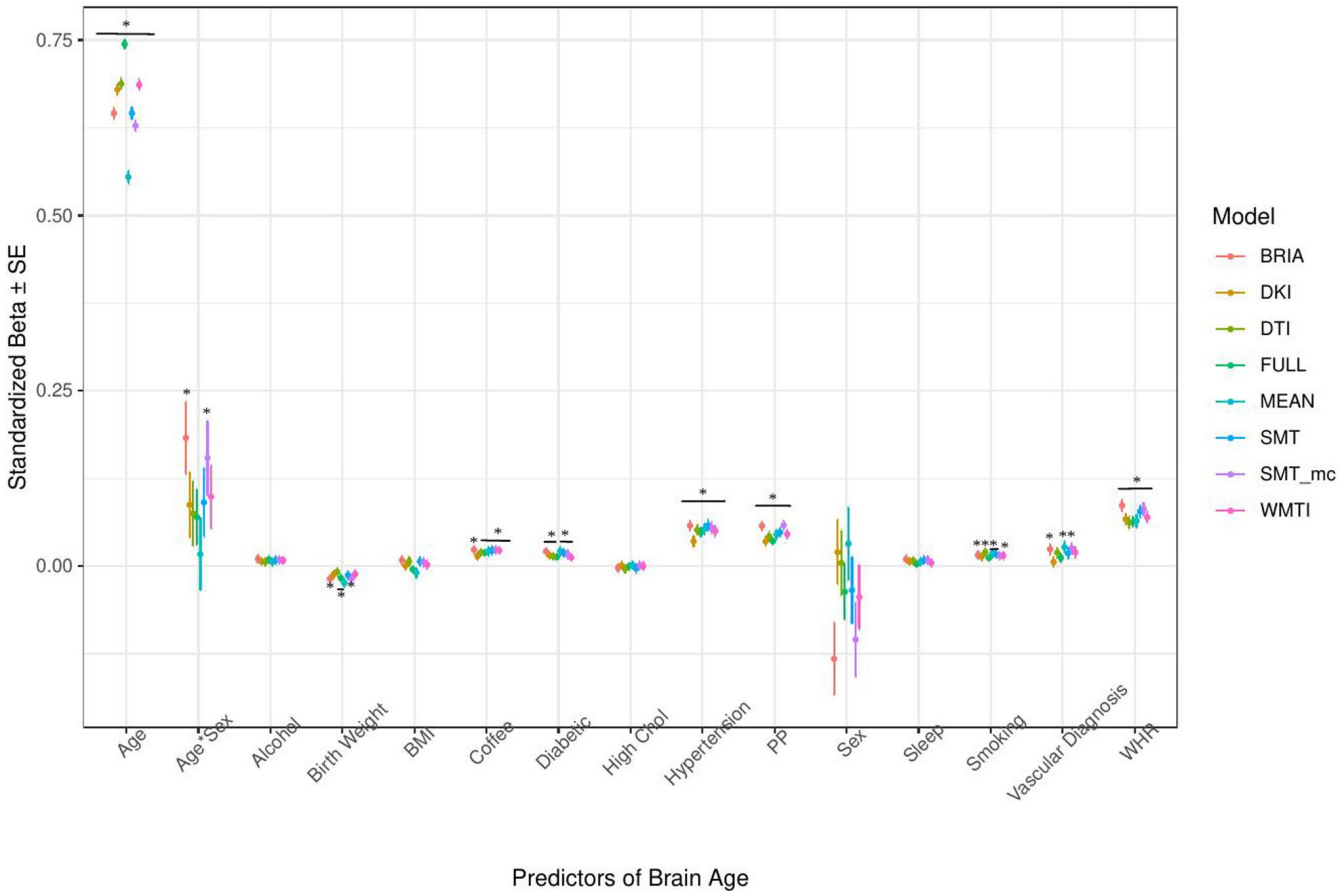
Health model predictors’ standardized beta-values with standard error. *Indicates Bonferroni-corrected *p* < 0.05.

**Figure 6:**
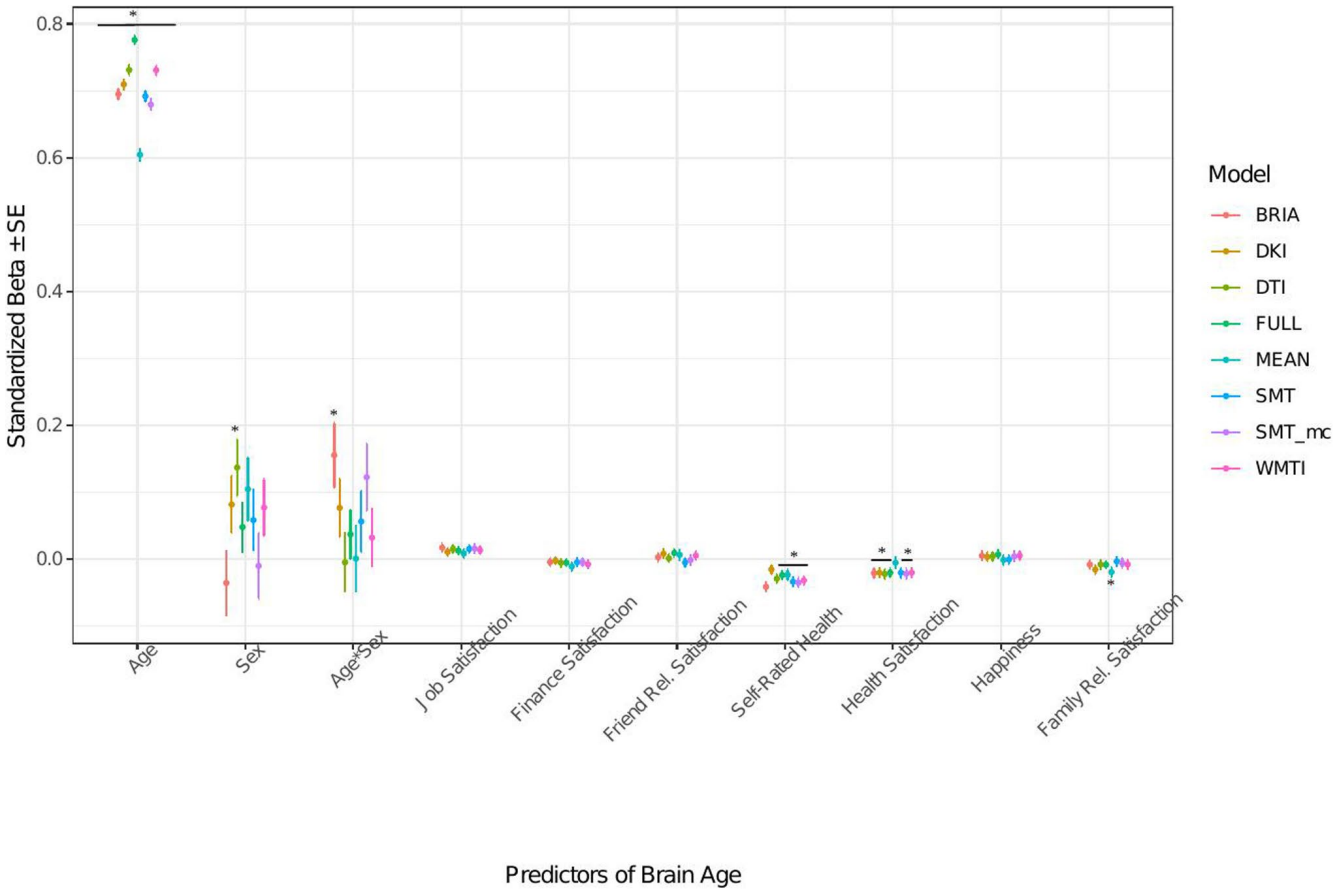
Well-being model predictors’ standardized beta-values with standard error. *Indicates Bonferroni-corrected *p* < 0.05.

**Figure 7:**
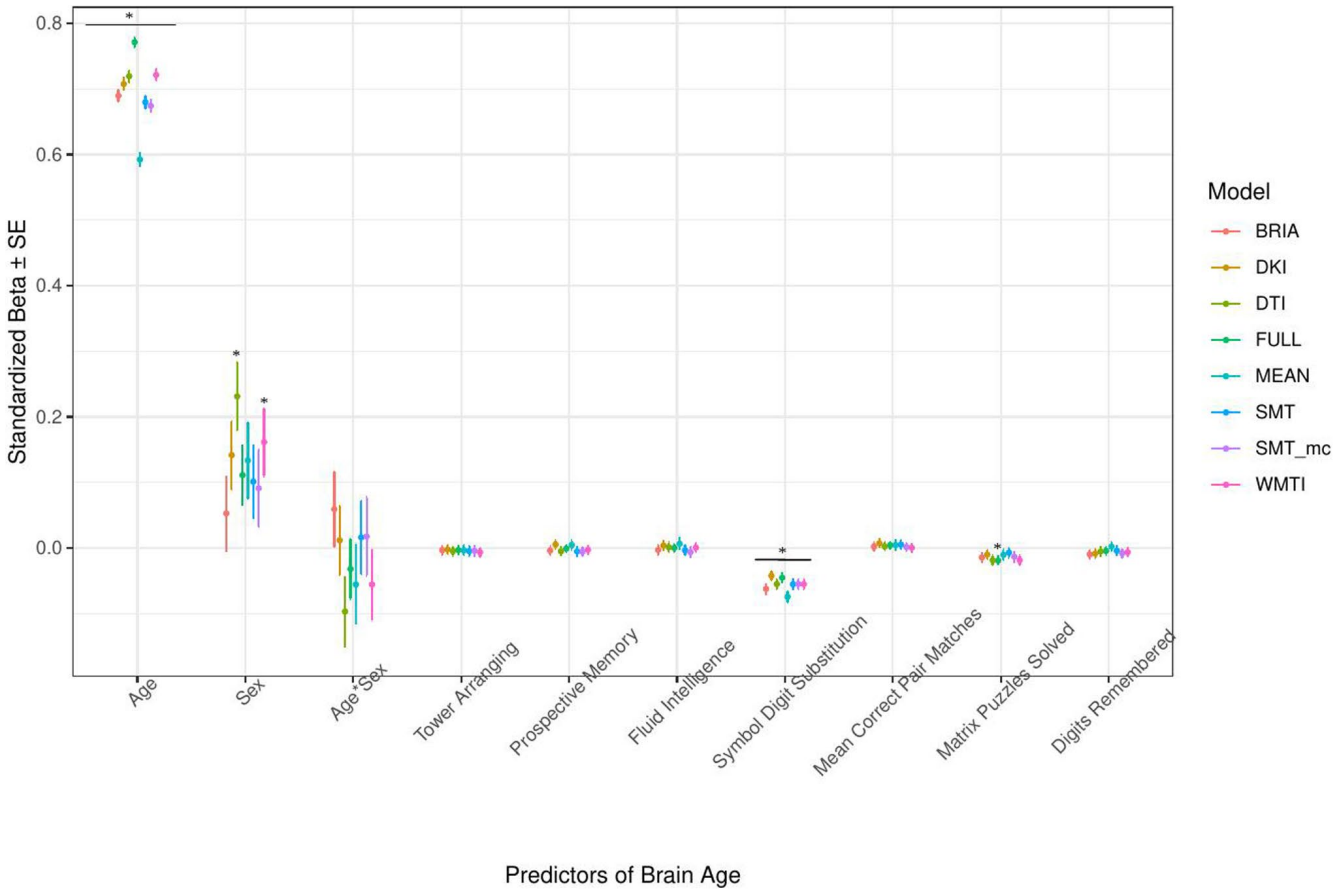
Cognition model predictors’ standardized beta-values with standard error. *Indicates Bonferroni-corrected *p* < 0.05.

Across statistical and diffusion models, age was used as a control variable to correct for the mere reflection of age by brain age producing stable associations across models (1–5) for multimodal brain age (Figures 4–7). However, except for the life-satisfaction model, in contrast to the full multimodal model, the other diffusion approaches’ brain ages were negatively associated with age, giving another indication of overall poor model fit. Even more so, the effect of sex was dependent on the model, producing mixed effects with large uncertainty surrounding *β-*values, also in the age-by-sex interactions’ associations with brain age. Overall, bio-psycho-social factors were consistently associated with brain ages from different diffusion approaches, with the exception for sex (Figures 4–7).

#### 3.1.1. Sociodemographic factors’ associations with brain age

In the model including sociodemographic factors explaining brain age (see Figure 4 for the predictors), results were mixed for the significant predictors. Sex was a significant predictor for mean DKI, DTI, and WMTI (*ps* < 0.05), the age-by-sex interaction only for BRIA (*p* = 0.045), and ethnicity only for DKI (*p* = 0.012; Figure 4). Overall, only 95% confidence intervals of *β-*values for age and ethnicity were not consistently overlapping, indicating differential effects of these variables on brain age based on the underlying data. All other 95% confidence intervals surrounding coefficients’ *β-*values were overlapping across diffusion approaches, with expected strong age contributions predicting brain age.

#### 3.1.2. Health and lifestyle factors’ associations with brain age

Similarly, in the model including health and lifestyle factors explaining brain age (see Figure 5 for the predictors), significant health factors leading to higher brain age were WHR (*ps* < 0.001), pulse pressure (*ps* < 0.001), and hypertension (*ps* < 0.001). Evidence across diffusion approaches was mixed for the other predictors with smoking predicting brain age derived from BRIA, DTI, mean scores, SNT, and WMTI (*ps* < 0.05), diabetes diagnosis for all models except DKI and DTI (*ps* < 0.05), the diagnosis of at least one vascular disease for BRIA, mean scores, and mcSMT (*ps* < 0.02), and average daily cups of coffee for brain age estimates except the one based on BRIA (*ps* < 0.01), and the age-by-sex interaction for BRIA and mcSMT (*ps* < 0.04).

Interestingly, WHR was a stronger predictor of brain age in males than in females (Supplementary Figure S5). Practically, a WHR *β_unstd_*-value of, for example *β* = 4 would mean that for every 0.1 step change in WHR, the brain age can be expected to increase by 0.4 years (see Supplementary Figures S6–S9 for *β_unstd_*). Importantly, this association was controlled for age, as age is correlated with WHR at *r* = 0.14 and brain age at *r* = 0.80. Mean population values for WHR were found to be WHR < 1 (Molarius et al., 1999), with our sample corresponding with these estimates (*M*_WHR_ = 0.871 ± 0.088, min = 0.534, max = 1.472) with males having a higher WHR (*M*_WHR_ = 0.923 ± 0.064) than females (*M*_WHR_ = 0.817 ± 0.069).

BMI was potentially non-significant due to the model construction as the highly correlated WHR (Supplementary Figure S4) was a significant predictor of brain age, and BMI alone being a significant predictor of brain age (Table 2). Finally, higher birth weight was associated with lower brain age estimated from full and mean models, as well as BRIA and WMTI (*ps* < 0.02).

**Table 2:** Linear models relating multimodal brain age to bio-psycho-social factors.

Variable	Level or metric	Variable value	Brain age^1^/Age^1^	*N*^2^	Marginal *R*^2^ diff	Log Likelihood_diff_	*χ*^2^ *p*_diff_^4^	*β_raw_/β_std_*^3^	*p*_pred_^4^
Brain age	Mean ± SD	64.470 ± 5.946		32,174					
Demographics									
Scanner site	% Cheadle	57.559	63.6/63.6	18,519					
	% Newcastle	26.403	65.3/65.2	8,495					
	% Reading	15.913	66.1/66.2	5,120					
	% Bristol	0.124	67.0/67.2	40					
Sex	% Male	47.122	65.4/65.1	17,013				−1.07/0.09	0.025
	% Female	52.878	63.7/63.9	15,161					
Age	Mean ± SD	64.473 ± 7.614	64.5/64.5	32,174				0.62/0.79	<0.001
Socio-demographics									
Ethnicity	European	96.800	64.5/64.6	31,160	−1.8 × 10^−5^				
	Non-European	2.970	62.3/61.2	956	−1.8 × 10^−5^	4	0.007	−0.31/–0.01	185
	Prefer not to say	(0.180)	65.6/65.6	58					
Income^5^	% less £18 k	10.363	65.6/66.1	3,310	1.4 × 10^−4^				
	% £18 k-£30	24.253	65.6/66.5	7,747	1.4 × 10^−4^	11	<0.001	−0.28/–0.02	0.003
	% £30 k-£52 k	27.713	64.6/64.7	8,852	1.4 × 10^−4^	11	<0.001	−0.29/–0.02	<0.001
	% £52 k-100 k	21.586	62.9/61.7	6,895	1.4 × 10^−4^	11	<0.001	−0.18/–0.01	0.4
	% > £100 k	6.956	61.5/59.4	2,222	1.4 × 10^−4^	11	<0.001	−0.30/–0.01	0.075
	Do not know	3.040	67.2/68.0	972					
	Prefer not to say	6.086	65.6/66.8	1944					
Higher education	% Yes	49.326	64.2/63.9	15,870	5.2 × 10^−5^	1	0.177	5.3 × 10^−2^/–0.004	1
	% No	50.674	64.8/65.0	16,304	5.2 × 10^−5^	1	0.177		
Cognitive test scores									
Matrix puzzles solved	Mean ± SD	8.011 ± 0.500	64.8/64.7	21,755	6.8 × 10^−4^	27	<0.001	−0.08/–0.03	<0.001
Tower rearranging correct attempts	Mean ± SD	9.920 ± 2.123	64.8/64.7	21,587	5.0 × 10^−4^	17	<0.001	−0.04/–0.02	<0.001
Prospective memory	Mean ± SD	1.067 ± 0.397	64.3/64.3	30,300	−1.7 × 10^−6^	0	0.312	−0.05/–0.003	1
Fluid intelligence	Mean ± SD	6.631 ± 0.397	64.3/64.2	29,786	7.3 × 10^−4^	23	<0.001	−0.07/–0.02	<0.001
Digits remembered	Mean ± SD	6.675 ± 1.540	64.9/64.8	23,070	1.6 × 10^−4^	15	<0.001	−0.06/–0.02	0.002
Mean number of incorrect pair matches across trials	Mean ± SD	2.214 ± 1.279	63.9/63.8	20,770	2.9 × 10^−4^	3	0.014	−0.05/0.01	0.375
Life satisfaction^6^									
Job satisfaction	Mean ± SD	4.511 ± 0.863	62.7/61.6	18,399	−5.5 × 10^−6^	1	0.494	0.02/0.003	1
Financial satisfaction	Mean ± SD	4.714 ± 0.828	64.4/64.5	31,909	2.6 × 10^−4^	10	<0.001	−0.10/0.003	<0.001
Health satisfaction	Mean ± SD	4.470 ± 0.766	64.5/64.5	31,911	0.001	52	<0.001	−0.26/0.003	<0.001
Overall health rating	Mean ± SD	3.030 ± 0.630	64.5/64.5	31,934	0.001	66	<0.001	−0.36/–0.04	<0.001
Family relation satisfaction	Mean ± SD	4.814 ± 0.846	64.4/64.4	31,737	2.7 × 10^−4^	17	<0.001	−0.10/0.003	<0.001
Friend relationship satisfaction	Mean ± SD	4.784 ± 0.846	64.4/64.4	31,640	2.0 × 10^−6^	0	0.485	−0.02/0.003	1
Happiness	Mean ± SD	4.542 ± 0.686	64.5/64.5	31,884	1.2 × 10^−4^	3	0.011	−0.07/–0.008	0.275
Health and lifestyle factors									
BMI	Mean ± SD	26.319 ± 4.269	64.4/64.4	31,052	5.9 × 10^−4^	41	<0.001	0.04/0.03	<0.001
Pulse pressure	Mean ± SD	60.027 ± 14.540	64.3/64.3	28,184	0.002	66	<0.001	0.02/0.05	<0.001
WHR	Mean ± SD	0.872 ± 0.088	64.4/64.4	31,138	0.004	129	<0.001	4.86/0.07	<0.001
Smoking	% Yes	2.629	63.0/61.4	838	7.5 × 10^−5^	4	0.005	0.34/0.01	0.15
	% No	97.374	64.5/64.5	31,033	7.5 × 10^−5^	4	0.005		
Diabetes	% Yes	1.688	66.6/66.1	543	5.1 × 10^−4^	22	<0.001	0.99/0.02	<0.001
	% No	98.312	64.4/64.4	31,631	5.1 × 10^−4^	22	<0.001		
Hypertension	% Yes	19.680	66.7/66.9	6,332	0.002	151	<0.001	0.86/0.06	<0.001
	% No	80.320	63.9/63.9	25,842	0.002	151	<0.001		
High cholesterol	% Yes	12.202	66.9/68.0	3,926	−7.4 × 10^−5^	18	<0.001	0.26/0.01	<0.001
	% No	83.798	64.1/64	28,248	−7.4 × 10^−5^	18	<0.001		
Vascular diagnosis	% Yes	22.726	66.7/67.1	7,312	0.002	136	<0.001	0.78/0.06	<0.001
	% No	87.274	63.8/63.7	24,862	0.002	136	<0.001		
Birth weight (kg)	Mean ± SD	3.358 ± 0.619	63.7/63.3	19,409	3.0 × 10^−4^	10	<0.001	−0.18/–0.02	<0.001
Daily coffee intake (cups)	Mean ± SD	2.065 ± 1.815	64.5/64.5	31,973	3.0 × 10^−4^	20	<0.001	0.06/0.02	<0.001

Linear models were used to observe each of the bio-psycho-social variables of interest in a model of [brain age ~ age + sex + age*sex + bio-psycho-social variable] with scanner site as a random factor. The model was then compared to a baseline model of [brain age ~ age + sex + age*sex] with scanner site as a random factor, and hence, *R*^2^ values refer to variance uniquely contributed by the single bio-psycho-social variable when added to the baseline model, referring to the marginal *R*^2^. For sex and age, fixed effects, and site, the random effect are being presented from the baseline model. ^1^These values differ because of missing data in the associated bio-psycho-social factors and will be influenced by the model’s age bias. ^2^Sample sizes differ due to missing data in the bio-psycho-social variables. ^3^β_raw_/β_std_ are the raw and standardized *β* values of the bio-psycho-social variables of interest. ^4^p_pred_ refers to the Bonferroni-corrected (multiplied by the number of bi-variate tests = 25) *p*-values of the predictors/bio-psycho-social variables’ β, *p*_diff_ refers to the differences between baseline model and the respective model including the bio-psycho-social variable of interest using χ^2^ test. ^5^Likert-type scales were applied for self-rating scales ranging from 1 “extremely unhappy” to 6 “extremely happy” with the exception of overall health ranging from 1 “poor” to 4 “excellent.”

Generally, 95% confidence intervals around coefficients’ *β*-values were overlapping across models indicating no significant differences in *β*-values across diffusion approaches. As a control, we ran the same model without WHR as predictor, due to its high correlation with BMI, rendering BMI as significant predictor across diffusion approaches’ brain ages except the mean model (*β*s > 0.01, *p*s < 0.004), also showing now clearer evidence for higher brain age when smoking (*p*s < 0.05), with other predictors unchanged (Supplementary Figure S11). Furthermore, leaving out hypertension, being a substrate of blood pressure, did not lead to changes in the model (Supplementary Figure S12). For both models, variance explained is slightly reduced compared to the models including the respective variables, making the reduced models significantly different (*p*s < 0.001) from the full health models (Supplementary Tables S8, S9).

#### 3.1.3. Life satisfaction factors’ associations with brain age

When modeling brain age from life satisfaction (see Figure 6 for the predictors), self-rated health was a significant predictor of all brain age estimates except for DKI brain age (*ps* < 0.05) and health satisfaction for all brain age estimates except the mean model’s brain age (*ps* < 0.02). Only the 95% confidence intervals of *β-*values for age do not overlap across models (with the mean model having the largest *β* and full model the smallest *β-*value for age). All other 95% confidence intervals around coefficients’ *β-*values overlap across models indicating no significant differences in *β-*values across diffusion approaches.

Perceived health is moderately correlated with health satisfaction and was left out in a control model resulting in a slightly stronger effect of health satisfaction and significantly worse performing model (*ps* < 0.001; Supplementary Figure S12 and Supplementary Tables S8, S9). Differently, when leaving out happiness as being correlated with several variables the model remains unaffected (*ps* > 0.23; Supplementary Figure S13 and Supplementary Tables S8, S9).

#### 3.1.4. Cognitive factors’ associations with brain age

The only cognitive factor explaining brain age across all models was symbol digit substitution (*ps* < 0.001; Figure 7). Matrix puzzles solved was only a significant predictor for the full multimodal brain age (*p* = 0.014), and sex only for DTI and WMTI (*ps* < 0.02). Confidence intervals around coefficients’ *β-*values are overlapping across models indicating no significant differences in *β-*values across diffusion approaches. Fluid intelligence and matrix puzzles are highly correlated and hence, matrix puzzles were left out in a quality control model, not significantly affecting the structure of most models (Supplementary Figure S14 and Supplementary Tables S8, S9).

#### 3.1.5. Follow-up: quality control and bivariate relationships of multimodal brain age and bio-psycho-social factors

Due to the strong variability in sex *β-*values across models (Figures 2, 4–6), we also ran the described analyses separately for males and females showing some differences in model performance. For example, bio-psycho-social models explained a differential of between 1 and 4% of conditional variance for males (Supplementary Table S10) and differences in contributions of the different models’ predictors, predictors’ *β-*values being generally higher for males (Supplementary Figure S2). Overall, quality checks show small levels of multicollinearity, and that each predictor contributes individual to the models (Supplementary Figures S2–S10 and Supplementary Tables S8, S9), supporting assumptions about the robustness of the utilized models, as well as that simply adding all variables together saturates the model leading to lower model performance than at baseline across brain ages based on different diffusion approaches with a differential in marginal *R*^2^ = 3.38%.

Finally, for a better understanding of bivariate relationships, Table 2 gives an overview of brain age calculated from combined single and multi-shell diffusion data in relation to the observed bio-psycho-social factors. Strongest standardized associations when adding single factors to a model explaining brain age from age were found for WHR (*β_std_* = 0.07, *p* < 0.001), PP (*β_std_* = 05, *p* < 0.001), and overall health rating (*β_std_* = −0.04, *p* < 0.001), and health satisfaction (*β_std_* = −0.03, *p* < 0.001). Strongest brain age group differences were found for sex (*β_std_* = −0.09, *p* = 0.001), diabetes (*β_std_* = 0.02, *p* < 0.001), and hypertension (*β_std_* = 0.06, *p* < 0.001).

## 4. Discussion

We assessed the influence of various bio-psycho-social variables on brain age estimated from different diffusion approaches (and their combinations). As predicted, linear mixed effects models showed that bio-psycho-social variables uniquely explain a small proportion of brain age variability consistently across models, and estimates overlap for most predictors. Health and lifestyle factors were most indicative of brain age. However, differences in brain age variance explained between bio-psycho-social models and diffusion approaches were small. Significant predictors of brain age were job satisfaction, health satisfaction, WHR (and to a lesser extent BMI when excluding WHR as a predictor), diabetes, hypertension, any vascular diagnosis, daily coffee consumption, smoking, birth weight, matrix puzzles, and symbol digit substitution performance. Our findings indicate that brain age estimates derived from different diffusion approaches relate similarly to the examined bio-psycho-social factors. This is an important finding as it reveals that different WM characteristics share common aging associations, which are detailed by bio-psycho-social factor associations. The presented diffusion approaches are based on different theoretical assumptions for deriving a set of WM features. For example, DTI and DKI metrics are usually quite sensitive to a broad range of WM changes due to their integrative nature of the scalar metrics (Basser et al., 1994; Jensen et al., 2005), i.e., DTI’s FA or DKI’s MK allow one to detect and localize the WM changes but not to explain their origins. In turn, dMRI approaches such as SMTmc or BRIA offer several metrics potentially allowing us to bind WM architecture with their predictive power (Kaden et al., 2016a,b; Reisert et al., 2017). For example, the intra-axonal water fraction appearing in both models might correlate with axonal density and axon diameter (Jelescu et al., 2020). Consequently, the metric provides information about WM maturation associated with aging leading to similar associations with age, aging and aging-related variables as DTI/DKI models. This encourages the usage of both conventional and advanced diffusion approaches when examining the relationship of bio-psycho-social factors and WM. Particularly, the application of dMRI approaches with more accurate assumptions around biophysical processes such as a ratio between intra-and extra-axonal diffusivities, permeability and other features offers various opportunities to investigate aging and associated diseases.

### 4.1. Explaining brain age from bio-psycho-social factors

Recent research has made a strong case for the conjunction effects of various bio-psycho-social factors in explaining general health (Lehman et al., 2017). Applied to brain age, for example, cardiometabolic effects have been shown to influence brain age (Beck et al., 2022a,b). However, assessments of how much of the variance explained in brain age above and beyond age, sex, age-by-sex interaction, and scanner site have not been described in the literature. We find close-to-zero added brain age variance explained by models including single bio-psycho-social variables (Table 2). Principal components of the health and lifestyle, life satisfaction, socio-demographics, and cognitive ability variables also added only small levels of brain age variance explained to the baseline model. A comparably larger proportion of brain age variance (*R*^2^ < 4%) is uniquely explained by health and lifestyle, life satisfaction, socio-demographics, and cognitive ability variables underlying the principal components (Figure 2 and Supplementary Table S7). These results suggest to include the different bio-psycho-social variables as predictors in order to explain brain age and the full covariance structure rather than using components which reduces the covariance matrix.

Health and lifestyle factors explained most brain age variance when added to the baseline model, followed by life satisfaction, and sociodemographic factors. Adding cognitive scores to the baseline model decreased brain age variance explained by the model (Figure 2). This suggests that biological and psychological factors are more influential than demographic factors. In turn, the observed bio-psycho-social factors are not independent of each other. Thus, we assume that bio-psycho-social factors contribute to explainations of brain age conjunctively. Additionally, we revealed that the added variance explained was small across models. A potential reason for small added R^2^ values might lay in multiple confounder effects and heterogeneity in effects across covariate levels (Table 2 fallacy, Westreich and Greenland, 2013). Importantly, the added brain age variance explained is not just an effect of adding predictors randomly to the model, which rather decreases the variance explained, as shown when adding all bio-psycho-social variables to the model. Hence, it seems more sensible to employ models incorporating several compared to single domain-specific variables to explain brain age. However, our results also indicate that a large part of the variance in brain age cannot be explained by our proposed bio-psycho-social models. Whether this unexplained variance is due to actual biologically founded individual differences, or the characteristics of brain age, for example, how the metric is being estimated (de Lange et al., 2022), remains unclear. BAG might also be rather static and indicated by constants such as genetic architecture and birth weight (Vidal-Pineiro et al., 2021). This would explain the smaller influence of more variable bio-psycho-social variables. Strong deviations from the norm, for example, due to atrophy will also have a strong influence on brain age (Kaufmann et al., 2019). Hence, for diseases impacting brain structure, brain age can be a useful indicator of health status (Kaufmann et al., 2019). Potentially, the health and lifestyle factors which are most likely to impact brain structure are therefore also more predictive of brain age than other bio-psycho-social variables (Figures 4–7). While our models failed to explain larger proportions of the variance of brain age, there are various interesting phenotype associations within these models which will be discussed in the following.

#### 4.1.1. The importance of age, sex, and ethnicity

Usually, age, sex, and at times, scanner site, are used as covariates for brain age-phenotype associations as they are expected to influence various phenotypes (Jirsaraie et al., 2022). As brain age reflects chronological age, age also explains most of the brain age variance (Figures 4–7). We also find that the effects of sex and the sex-age interaction were highly variable across diffusion models predicting brain age with sex and the sex-age interaction being mostly non-significant predictors across diffusion models (Figures 4–7). Nevertheless, brain age does significantly differ between sexes (Sanford et al., 2022; Subramaniapillai et al., 2022), and we cannot exclude sex difference in WM microstructure. These relationships might also lead to differences in WM brain ages between sexes. Furthermore, models were more predictive of bio-psycho-social factors in males than females (Supplementary Table S2 and Supplementary Figure S2). Where the influence of sex changes based on the model construction, while potentially also influencing the model (Figures 4–7 and Supplementary Figures S5–S8). Some of the observed sex differences might be based on anatomical features, such as higher intracranial volume in males and different sex-specific aging (Eikenes et al., 2022). Brain age was differentially sensitive to ethnicity dependent on the approach it was calculated on (Figures 4–7), with these differences being influenced by sex (Supplementary Figure S2). A previous study showed that being a UK immigrant might influence brain age estimates (Leonardsen et al., 2022). Potentially, genetic contributions to brain age both estimated from T1-weighted (Ning et al., 2020; Vidal-Pineiro et al., 2021) and dMRI data (Salih et al., 2021) also have a connection with the mentioned brain age differences by sex and ethnicity. However, the causal structure of sex and ethnicity differences in brain age estimates requires further investigation.

Previous research has shown the effects of sex on metrics derived from conventional and advanced diffusion approaches, such as BRIA, DKI, DTI, NODDI, RSI, SMT, SMT mc, and WMTI (Beck et al., 2021; Eikenes et al., 2022). While a systematic assessment of sex-related effects on diffusion metrics from both conventional and advanced dMRI approaches from voxel-to-whole-brain averages over the lifespan is yet to be established, different studies presented sex-related developmental trajectories in the structural connectome in children (Ingalhalikar et al., 2014), and sex related WM changes during aging (Hsu et al., 2008). Furthermore, sex differences in aging reflected in WM microstructure can be expected due to menopause and cascading biological processes, affecting both brain and body systems in various ways (Barth and de Lange, 2020; Mosconi et al., 2021; Lohner et al., 2022). Hence, developmental trajectories differing between males and females can be expected which makes sex-separated analyses useful to providing important additional information (e.g., as in Subramaniapillai et al., 2022). To which extend this applies to ethnicity requires further research. Hence, further research is required to delineate the underlying causal structure of sex and ethnicity to explain their highly variable associations with brain age.

#### 4.1.2. Health and lifestyle factors

Interestingly, while the health and lifestyle factors models explained only a small proportion of the brain age variance, most of its predictors were significant. Furthermore, these predictors are generally only weakly correlated (Supplementary Figure S5), but when added in conjunction explaining more variability in brain age than on their own (compare Table 2 and Figure 5). To a certain degree, this is not surprising, due to dependencies between these predictors. For example, WHR, being the strongest predictor of brain age (see Figure 5), shows a clear relationship with pulse pressure (Supplementary Figure S5). For the extreme cases, this is expressed in a well-established relationship between obesity and hypertension (Kotsis et al., 2010) or any vascular diagnosis (Mathew et al., 2008). This is reflected in brain age, where minimum and maximum values show that there is an expected difference of up to 4 years in brain age between those with lowest compared to highest WHR, or a 2.4-years brain age difference between mean and maximum WHR. Interestingly, blood pressure is expected to increase with age, and higher blood pressure is positively associated with BAG (Cherbuin et al., 2021). However, these effects were not exclusively driven by hypertension but across the spectrum of measured blood pressure values (Cherbuin et al., 2021). This was supported by our findings showing both an effect of pulse pressure and hypertension on brain age. These effects are not surprising, as hypertension has been suggested as one of the most important risk factors for various cerebrovascular complications such as cerebral small vessel disease and resulting cognitive impairments (Meissner, 2016; Forte et al., 2019).

Another aspect of high WHR and BMI is obesity increasing diabetes risk (Kahn et al., 2006). While the evidence for the direction of the effect of diabetes is mixed (Franke et al., 2013; Cole et al., 2018; Sone et al., 2022), we find participants with diabetes to show higher brain age than those without diabetes (Table 1 and Figure 5). Several complications within the central nervous system have been associated with diabetes, including morphological, electrophysiological, and cognitive changes, often in the hippocampus (Wrighten et al., 2009), just as WM lesions and altered metabolite ratios (van der Harten et al., 2006; Biessels and Reijmer, 2014), supporting the idea of higher brain age among those with diabetes. But also generally, the increase in risk of cardiovascular disease by WHR is mediated by BMI, systolic blood pressure, diabetes, lipids, and smoking (Gill et al., 2021). In relation to the brain, higher WHR has been generally associated with lower gray matter volume (Hamer and Batty, 2019; Gurholt et al., 2021), and higher WM brain age (Beck et al., 2022a,b; Subramaniapillai et al., 2022). Hence, to which extent high WHR accelerates brain aging requires further investigation, which might be particularly informative when observed in combination with other health and lifestyle variables (Hamer and Batty, 2019) and sex (Subramaniapillai et al., 2022).

Negative health consequences of smoking (Erhardt, 2009) are reflected in smoker’s cortex being thinner (Gurholt et al., 2021), and smokers’ brains being 1.5 years older on average than non-smokers’ brains (Table 2). Smoking is a known risk factor for cardiovascular health significantly increasing its mortality and inducing various negative downstream effects on health (Erhardt, 2009), with negative impacts on the reward system (Le Foll et al., 2022), repeatedly shown in rats (e.g., Gozzi et al., 2006; Kenny and Markou, 2006; Cao et al., 2013). It can hence be expected that both general and brain health are influenced by smoking, making it an important control variable in assessing brain age.

The findings for coffee on the other hand are mixed, suggesting coffee consumption to be generally positive for cardiovascular health and decreasing the risk of Parkinson’s disease, stroke, and Alzheimer’s (Nehlig, 2016). The consumption of higher doses of caffeine is, however, associated with smaller brain volume and an increased risk of dementia (Pham et al., 2022). Practically, the direct effect of the number of daily cups of coffee consumed is small in our study. It would require on average 10 cups of coffee daily for an increase of 0.6 years of brain age, fitting the observations made by Pham et al. (2022). It also remains unclear whether the effect of coffee consumption on brain age is rather mediated by third variables such as poor sleep and mental health downstream effects which show direct negative effects on health (Distelberg et al., 2017). Additionally, there are vulnerable groups in which caffeine can cause adverse effects such as people with hypertension (Higdon and Frei, 2006). We conclude that health and lifestyle factors function in synergy in influencing brain age.

#### 4.1.3. Health perception and satisfaction, and job satisfaction

We find significant assignations of self-rated health, friendship/relationship satisfaction, and job satisfaction with brain age. Self-assessments and self-rated scores are some of the fastest and easiest assessments. Yet, their reliability is under constant scrutiny, particularly when assessing health outcomes (e.g., Crossley and Kennedy, 2002; Reychav et al., 2019). In our study, self-rated overall health was a significant predictor of brain age, suggesting that asking participants about their health can be a useful preliminary assessment of different aspects of health. Self-rated health was additionally moderately correlated with health perception (Supplementary Figure S4), indicating both variables measure, to a certain degree, the same underlying phenomenon. However, self-rated health brain age associations were stronger and more variable across diffusion approaches’ brain ages (Figure 6). These associations support the idea of brain age is not only indicative of brain health, but also overall health (Kaufmann et al., 2019).

Lastly, there was a trend of individuals’ job satisfaction being associated with brain age (Figure 6). Conceptually, this would not be surprising as associations between wealth and health (e.g., Adler and Ostrove, 1999) as well as job (e.g., Faragher et al., 2013) and financial satisfaction and health (e.g., Hsieh, 2001) have already been investigated. However, in the case of our study, higher job satisfaction was also indicative of higher brain age. Potential reasons are speculative but might reflect the tendency of people engaged in their jobs to work long hours which has previously been related with various negative mental and physical health outcomes (Lim et al., 2010; Bannai and Tamakoshi, 2014). Nevertheless, the underlying mechanisms of the associations between these single items in their relationship with brain age require further investigation.

#### 4.1.4. Cognitive scores

Cognitive scores’ impact on brain age might be small in the current study, yet still important in general (Table 2). This might be due to the selection of the observed cognitive test scores, with many more possible tests to be included which are potentially more indicative of brain age, such as IQ (Elliott et al., 2021). Another opportunity lies in assessing associations of cognitive performance and brain age in clinical groups. For example, brain age has been found to be explanatory of symbol digit modality test scores in multiple sclerosis suggesting brain age as a biomarker for cognitive dysfunction (Denissen et al., 2022). Similar to such findings, we find a similarly sized effect of symbol digit substitution test scores in our healthy aging data (Figure 7). Associations of cognitive performance and brain age are also sensitive to sex. For example, the number of solved matrix puzzles showing an effect when analyzing males and females data together seemed to be a predictor of brain age only in females when analyzing females from males data separately (Supplementary Figure S2). The quality of these differences requires further investigation.

### 4.2. Variability in brain age-phenotype relationships

Imaging phenotypes derived from diffusion UKB data contribute to a small additional proportion of the variability in the obtained results. However, the presented comparison of *R*^2^ differences (Figure 3) underestimates the effects of single bio-psycho-social factors, and has to be interpreted with care, with cognitive function, life satisfaction, and health and lifestyle factors significantly adding to the baseline model (Figure 3). Yet, the used brain age estimation model might also introduce variability in brain age phenotype associations. Problematically, model evaluation metrics such as *R*^2^, MAE, or RMSE depend additionally on cohort-and study-specific data characteristics making brain age model comparison across the literature not straightforward (de Lange et al., 2022). Additionally, there are differences between models trained on voxel-level compared to region-averaged data. Deep learning models using voxel-level data reach age predictions errors as low as MAE = 2.14 years in midlife to late adulthood (Peng et al., 2021) or MAE = 3.90 years across the lifespan (Leonardsen et al., 2022) while explaining large proportions of variance in age (*R*^2^ > 0.90), whereas models trained on regional and global average measures predict age usually with larger error, MAE > 3.6 years, and/or lower variances explained *R*^2^ < 0.75 (de Lange et al., 2020a,b; Beck et al., 2021, 2022b; Rokicki et al., 2021; Korbmacher et al., 2022). However, Niu et al. (2019) showed that with different shallow and deep machine learning algorithms (ridge regression, support vector regressor, Gaussian process regressor, deep neural networks) high prediction accuracies (*R*^2^ > 0.75, MAE < 1.43) could be reached when using multimodal regional average data using a young sample with narrow age range. Nonetheless, the same database (UKB) is able to provide similar patterns of detected associations between brain age and used phenotypes by applying different samples, modalities, and methods to calculate brain age. For example, diabetes diagnosis, diagnosed vascular problems or place of birth (see Figure 4 in Leonardsen et al., 2022), hip circumference, trail-making tasks, and matrix pattern completion were significantly associated with brain age (see Table 5 in Cole, 2020). However, it remains unclear whether the differences in the findings are due to analysis degree of freedom, sample characteristics, or actual bio-physical manifestations. For instance, the underlying data used for brain age estimation can be based on different modalities, e.g., dMRI metrics, as in the present work, versus T1-weighted images in Cole (2020) and Leonardsen et al. (2022). We can assume that WM-derived brain age associations with bio-psycho-social factors are relatively stable across diffusion approaches (see Figures 2, 4–6). We used four mixed models grouping (a) demographics, (b) cognitive, (c) life satisfaction, and (d) health and lifestyle variables to predict brain age. In contrast, Cole (2020) predicted bias-adjusted brain age from simple linear models with sex, age, and age^2^ as covariates, and Leonardsen et al. (2022) observed similar associations for uncorrected brain age predicted from the respective phenotype and age and sex as covariates. However, bio-psycho-social variables are likely to interact in a complex pattern when explaining variables such as brain age. If we add only single bio-psycho-social variables, such as waist-to-hip-ratio, to a baseline model and then compare the two models, the differences in variance explained are small. Adding blocks of meaningfully related variables leads to stronger increases in Brian age variance explained (compare Table 2 and Supplementary Table S7). In summary, there are various sources of variability in brain age prediction. Phenotype associations could encompass not only the underlying data but also researchers’ degree of freedom such as data selection, processing, and analysis.

## 5. Conclusion and future directions

Bio-psycho-social factors contribute similarly to explaining WM brain age across conventional and advanced diffusion MRI approaches when arranged as cognitive scores, life satisfaction, health and lifestyle factors, but not socio-demographics. Focusing on single predictors, health and lifestyle factors, WHR, birth weight, diabetes, hypertension, and related diagnoses, as well as smoking status and coffee consumption, were more predictive of brain age than cognitive and life satisfaction measures. Apart from health satisfaction and self-ratings, we found relationships of life satisfaction variables with brain age to be non-significant. Of the cognitive scores, only the digit substitution task performance was a significant predictor, which might be relevant in samples from midlife to old age. Furthermore, the influence of sex and ethnicity is largely variable suggesting the usage of sensible control mechanisms, such as separate analyses or exclusions in case of strongly imbalanced samples. We recommend future study designs taking observable interactions between the different bio-psycho-social effects into account. A potentially helpful guiding principle in the search for bio-psycho-social variables affecting brain age could be to focus on measures which are directly or indirectly related to or reflect pathology.

## Data availability statement

The datasets presented in this study can be found in online repositories. The names of the repository/repositories and accession number(s) can be found in the article/Supplementary material.

## Ethics statement

The studies involving human participants were reviewed and approved by National Health Service National Research Ethics Service (ref 11/NW/0382). The patients/participants provided their written informed consent to participate in this study.

## Author contributions

MK: study design, software, formal analysis, visualizations, project administration, writing –original draft, and writing – review and editing. TG: writing – review and editing. A-ML and DM: software, writing – review and editing. AL: funding acquisition. EE: writing – review and editing, and funding acquisition. DB: writing – review and editing. OA: writing – review and editing, and funding acquisition. LW: writing – review and editing, and funding acquisition. IM: supervision, study design, data pre-processing and quality control, writing – review and editing, and funding acquisition. All authors contributed to the article and approved the submitted version.

## Funding

This research was funded by the Research Council of Norway (#223273); the South-Eastern Norway Regional Health Authority (#2022080); and the European Union’s Horizon2020 Research and Innovation Programme (CoMorMent project; Grant #847776).

## Conflict of interest

OA has received a speaker’s honorarium from Lundbeck and is a consultant to Coretechs.ai.

The remaining authors declare that the research was conducted in the absence of any commercial or financial relationships that could be construed as a potential conflict of interest.

## Publisher’s note

All claims expressed in this article are solely those of the authors and do not necessarily represent those of their affiliated organizations, or those of the publisher, the editors and the reviewers. Any product that may be evaluated in this article, or claim that may be made by its manufacturer, is not guaranteed or endorsed by the publisher.

## Footnotes

^1^https://github.com/NYU-DiffusionMRI/DESIGNER

^2^https://github.com/ekaden/smt

^3^https://bitbucket.org/reisert/baydiff/src/master/

^4^www.r-project.org/
